# Acute changes in serum and skeletal muscle steroids in resistance-trained men

**DOI:** 10.3389/fendo.2023.1081056

**Published:** 2023-04-03

**Authors:** Felipe C. Vechin, Jakob L. Vingren, Guilherme D. Telles, Miguel S. Conceicao, Cleiton A. Libardi, Manoel E. Lixandrao, Felipe Damas, Telma F. Cunha, Patricia C. Brum, Luiz A. Riani, Carlos Ugrinowitsch

**Affiliations:** ^1^ School of Physical Education and Sport, University of São Paulo, São Paulo, Brazil; ^2^ Department of Kinesiology, Health Promotion, and Recreation, University of North Texas, Denton, TX, United States; ^3^ MUSCULAB - Laboratory of Neuromuscular Adaptations to Resistance Training, Department of Physical Education, Federal University of São Carlos, São Carlos, Brazil

**Keywords:** intracrine, steroidogenesis, intracrinology, strength exercise, measurement error

## Abstract

**Introduction:**

Resistance exercise can significantly increase serum steroid concentrations after an exercise bout. Steroid hormones are involved in the regulation of several important bodily functions (e.g., muscle growth) through both systemic delivery and local production. Thus, we aimed to determine whether resistance exercise-induced increases in serum steroid hormone concentrations are accompanied by enhanced skeletal muscle steroid concentrations, or whether muscle contractions per se induced by resistance exercise can increase intramuscular steroid concentrations.

**Methods:**

A counterbalanced, within-subject, crossover design was applied. Six resistance-trained men (26 ± 5 years; 79 ± 8 kg; 179 ± 10 cm) performed a single-arm lateral raise exercise (10 sets of 8 to 12 RM - 3 min rest between sets) targeting the deltoid muscle followed by either squat exercise (10 sets of 8 to 12 RM - 1 min rest) to induce a hormonal response (high hormone [HH] condition) or rest (low hormone [LH] condition). Blood samples were obtained pre-exercise and 15 min and 30 min post-exercise; muscle specimens were harvested pre-exercise and 45 min post-exercise. Immunoassays were used to measure serum and muscle steroids (total and free testosterone, dehydroepiandrosterone sulfate, dihydrotestosterone, and cortisol; free testosterone measured only in serum and dehydroepiandrosterone only in muscle) at these time points.

**Results:**

In the serum, only cortisol significantly increased after the HH protocol. There were no significant changes in muscle steroid concentrations after the protocols.

**Discussion:**

Our study provides evidence that serum steroid concentration increases (cortisol only) seem not to be aligned with muscle steroid concentrations. The lack of change in muscle steroid after protocols suggests that resistance-trained individuals were desensitized to the exercise stimuli. It is also possible that the single postexercise timepoint investigated in this study might be too early or too late to observe changes. Thus, additional timepoints should be examined to determine if RE can indeed change muscle steroid concentrations either by skeletal muscle uptake of these hormones or the intramuscular steroidogenesis process.

## Introduction

Steroid hormones are involved in the regulation of several important bodily functions (e.g., inducing male secondary characteristics, regulating steroidogenesis, retaining body nitrogen, and muscle growth) through both systemic delivery and local production. Systemic steroids permeate plasma membranes and bind to their receptor, and these complexes are subsequently translocated to the nucleus, where they bind to DNA and perform their main biological functions (1–3). In addition, steroid hormones can be locally produced (intracrine) *via* steroidogenesis in specific cells, including skeletal muscle cells. For instance, the steroid hormone testosterone is primarily produced in Leydig cells in the testicles of men, and cortisol is produced in the cells of the zona fasciculata in the adrenal cortex. However, skeletal muscle cells also have steroidogenesis potential (4, 5), as they express the enzymes that convert cholesterol to a range of steroids, including testosterone, dehydroepiandrosterone (DHEA), dihydrotestosterone (DHT), and cortisol (5–10). Although serum steroid hormones can permeate the sarcolemma and perform their biological function within the myonuclei, evidence supporting that their role, at least partially, in stress-induced responses in skeletal muscles is scarce.

An interesting model to test stress-induced responses in skeletal muscle is the use of resistance exercise (RE), as it can significantly increase serum steroid concentrations (e.g., DHEA, DHT, testosterone, and cortisol) for 15 minutes or more after an exercise bout (11–15). This higher availability of serum steroid hormones could increase intramuscular steroid hormone concentrations through increased permeation. Additionally, exercise-induced increases in serum steroids after an RE bout can increase the expression of steroid receptors over time in skeletal muscle cells (2, 16, 17), further increasing the potential biological effects of elevated hormonal concentrations in these cells. Mechanical stretch (e.g., muscle contractions) per se appears to increase the mRNA levels of steroidogenic enzymes in muscle cells (18), which could allow for additional synthesis of steroid hormones intramuscularly. Some authors showed that steroid concentrations and steroidogenic enzyme expression in human skeletal muscle increased after several RE bouts in older individuals (5). Nevertheless, RE-induced muscle steroidogenesis data are equivocal in human trials (16, 19). Thus, studies are needed to determine whether RE-induced increases in serum steroid hormone concentrations or intramuscular steroidogenesis *per se* affect intramuscular steroid concentrations.

Based on the aforementioned literature, the purpose of the current study is to investigate whether RE-induced increases in serum steroid hormone concentrations (total and free testosterone, dehydroepiandrosterone sulfate, and cortisol) are accompanied by elevated skeletal muscle steroid concentrations (total testosterone, dehydroepiandrosterone sulfate, dihydrotestosterone, and cortisol), or whether muscle contractions per se induced by RE can increase intramuscular steroid concentrations. Our hypotheses are (1) the RE-induced increase in serum steroid hormone concentrations is accompanied by elevated skeletal muscle steroid concentrations, and (2) muscle contractions per se induced by RE can induce increases in intramuscular steroid concentrations.

## Materials and methods

### Participants

Six young men (age, 26 ± 5 years; weight: 79 ± 8 kg; height: 179 ± 10 cm; BMI: 25 ± 2 kg.m^2-1^) who had been performing resistance exercises at least 3 times a week, including the back squat, for at least 3 years volunteered to participate in this study. To avoid any influence on the dependent variable due to unfamiliar stimuli, the study`s exercise protocols and the subjects’ regular training routines had similar exercises and training session volumes. Volunteers with a history of anabolic steroid use, chronic joint pain, and skeletal muscle injuries were excluded. The Institutional Review Board approved the experimental protocol. The study followed the latest revision of the Declaration of Helsinki. Participants were informed about the risks and benefits associated with the protocol before providing written informed consent.

### Experimental design

A counterbalanced (i.e., exercise protocol and limb dominance), within-subject, crossover design was used in the present study. The participants attended three laboratory visits. In the first visit, subjects were screened about their training experience and routines, daily food intake (with a three-day dietary record) and anabolic steroid use. Participants were instructed to avoid exercising for 72 hours before each laboratory visit and how to complete the dietary records. In the second and third visits, participants performed one of the assigned exercise sessions: low hormone RE session – LH or and high hormone RE session – HH. Subjects were instructed to fast for 6 hours (except for water) before the exercise sessions. Blood samples were drawn pre-exercise and 15 min and 30 min post-exercise. Muscle specimens were harvested pre-exercise and 45 min post-exercise. Considering that in blood, RE-induced increases in steroid concentrations subside by 30 min after an exercise bout, hormones may have been absorbed by the target tissues or metabolized in the liver (20). Additionally, Muscle steroidogenesis capacity in resistance-trained athletes has been investigated previously. Vingren, Kraemer (19) showed no changes in the concentration of testosterone in skeletal muscle at 10 and 70 min after RE despite a robust acute RE-induced increase in serum total and free testosterone concentrations (2). However, the large interval between these two time points and the lack of data for other steroids could explain their findings. We therefore assessed muscle steroid concentrations (i.e., testosterone, DHEA, DHT and cortisol) 45 min after the LH and HH protocols.

Participants were instructed to maintain their normal eating habits and to not use nutritional supplements throughout the duration of the study. Macronutrient ingestion was replicated (based on their records) in the last meal before each exercise protocol.

### Resistance exercise protocols

Participants performed the single-arm lateral raise exercise (10 sets of 8 to 12 repetition maximum (RM) - 3 min rest interval between sets) on the LH and HH protocols. In the HH protocol, the single-arm lateral rise was followed by back squat exercise in the Smith machine (10 sets of 8 to 12 RM - 1 min rest interval between sets) after a 2-min rest. To maintain a consistent repetition range of 8-12 repetition maximum (RM), the exercise workload was adjusted through sets. In brief, squat exercise sought to produce high systemic stress, which should increase the concentration of several serum steroid hormones, including testosterone and cortisol, after the exercise bout (14). Conversely, the LH protocol should not increase serum steroid hormone concentrations, as the single-arm lateral raise exercise involves a small muscle mass and induces a low systemic stress. Thus, the direct RE-induced stress and muscle activation imposed on the deltoid muscle were similar between LH and HH protocols, but the systemic stress and associated stress response should be greater in the HH protocol.

### Blood collection and muscle biopsies

Four milliliters of blood was drawn from an antecubital vein and allowed to clot at room temperature. Next, the samples were centrifuged for 10 min, and the serum fractions were stored at -20°C for future analyses. Muscle specimens from the medial deltoid muscle were obtained through percutaneous muscle biopsy with manual suction. After local anesthesia [2–3 ml of 1% Xylocaine (lignocaine)], ~100 mg of muscle was collected *via* a small incision with a Bergstrom needle (21). The tissue was dissected free from connective tissue and blood, immediately frozen in liquid nitrogen, and stored at -80°C until analysis.

### Serum steroid analysis

Total (T) and free testosterone (FT) and cortisol (C) concentrations were determined by ELISA (Diagnostics Biochem Canada Inc. DBC. Ontario Canada). The dehydroepiandrosterone sulfate (DHEA-S) concentration was determined by radioimmunoassay (Diagnostics Biochem Canada Inc. DBC. Ontario Canada). All analyses were carried out in duplicate and according to the manufacturer´s instructions. The interassay precision (CV%) for T and FT, DHEA and C were 6.1%, 11.5%, 6.7% and 8.1%, respectively.

### Skeletal muscle steroid analysis

Muscle samples (~50 mg) were homogenized in a specific ice-cold RIPA buffer using a fast prep device (Sample preparation system 24. M.P. Biomedicals, Irvine, California. USA). Homogenized muscle samples were agitated and centrifuged at 4°C and 15,000 × g for 15 min. The supernatant was collected and analyzed for total protein concentration using the Bradford protocol. The total protein concentration was used for normalizing muscle steroid concentrations. Muscle testosterone (T), dehydroepiandrosterone (DHEA), dihydrotestosterone (DHT), and cortisol (C) concentrations were determined in duplicate using commercially available EIA kits (Cayman – Ann Arbor, Michigan. USA; ENZO Life Sciences – Farmingdale, NY. USA; IBL international – Hamburg, Germany; R&D Systems – Minneapolis, MN. USA, respectively). Specific dilutions with EIA buffers were used for each steroid. T, DHEA, DHT and C samples were diluted 200, 20, 10, and 200 times, respectively. The interassay precision (CV%) for T, DHEA, DHT and C were 3.8%, 9.0%, 6% and 7.0%, respectively.

### Statistical analysis

Data normality was verified with the Shapiro-Wilk test. A mixed model was used for each dependent variable (serum and local steroids), with group (LH and HH) and time as fixed factors and subjects as a random factor. In the occurrence of a significant *F*-ratio, we used Tukey’s adjustments for pairwise comparisons. The significance level was defined as *P*< 0.05, and the data are presented as the mean ± standard deviation. To deal with missing data in small sample size we used the Kenward-Roger Degrees of Freedom Approximation (20). Statistical analyses were performed in SAS version 9.3 for Windows (SAS Institute Inc., Cary, NC). Due to the limited sample size and the need to discuss the data with extra caution, we used the coefficient of variation (CV) based on typical error (TE) (18). The intrasubject coefficient of variation (CV) was calculated as $CV=\left(\frac{TE}{mean}\right)\times 100$ , where the mean was calculated from all baseline values. The typical error (TE) was calculated as $TE=(SD\;of\;B_{1}-B_{2})/\sqrt{2}$ . SD stands for standard difference, B_1_ and B_2_ stand for baseline 1 and baseline 2.

## Results

**Figure 1:**
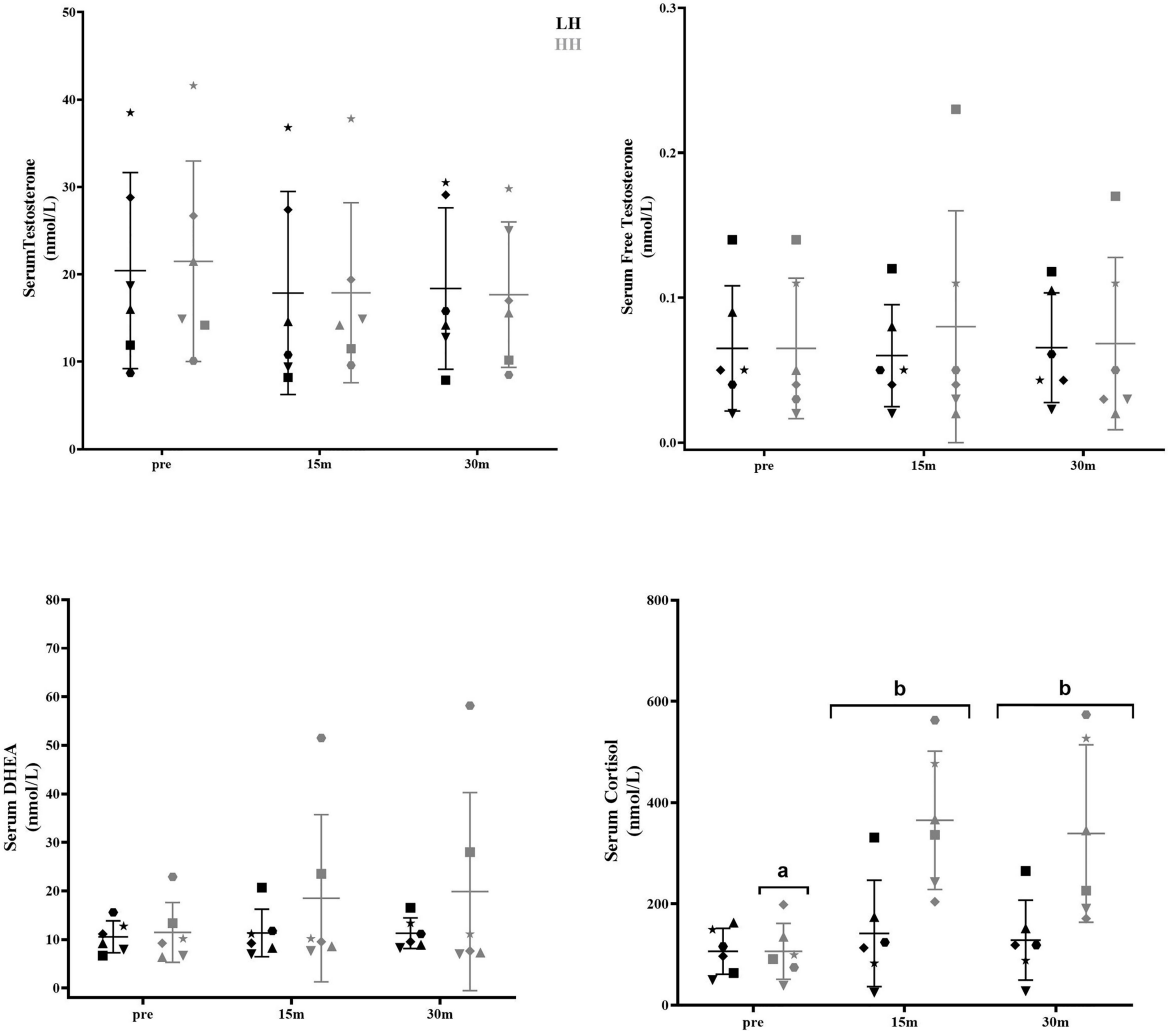
Serum steroid hormone concentrations pre-LH and HH protocols and at 15 min and 30 min postexercise. (a) Significantly different from corresponding 15-min and 30-min postexercise. (b) Significantly different from LH. Values are presented as the mean and SD. Each symbol represents one subject.

**Figure 2:**
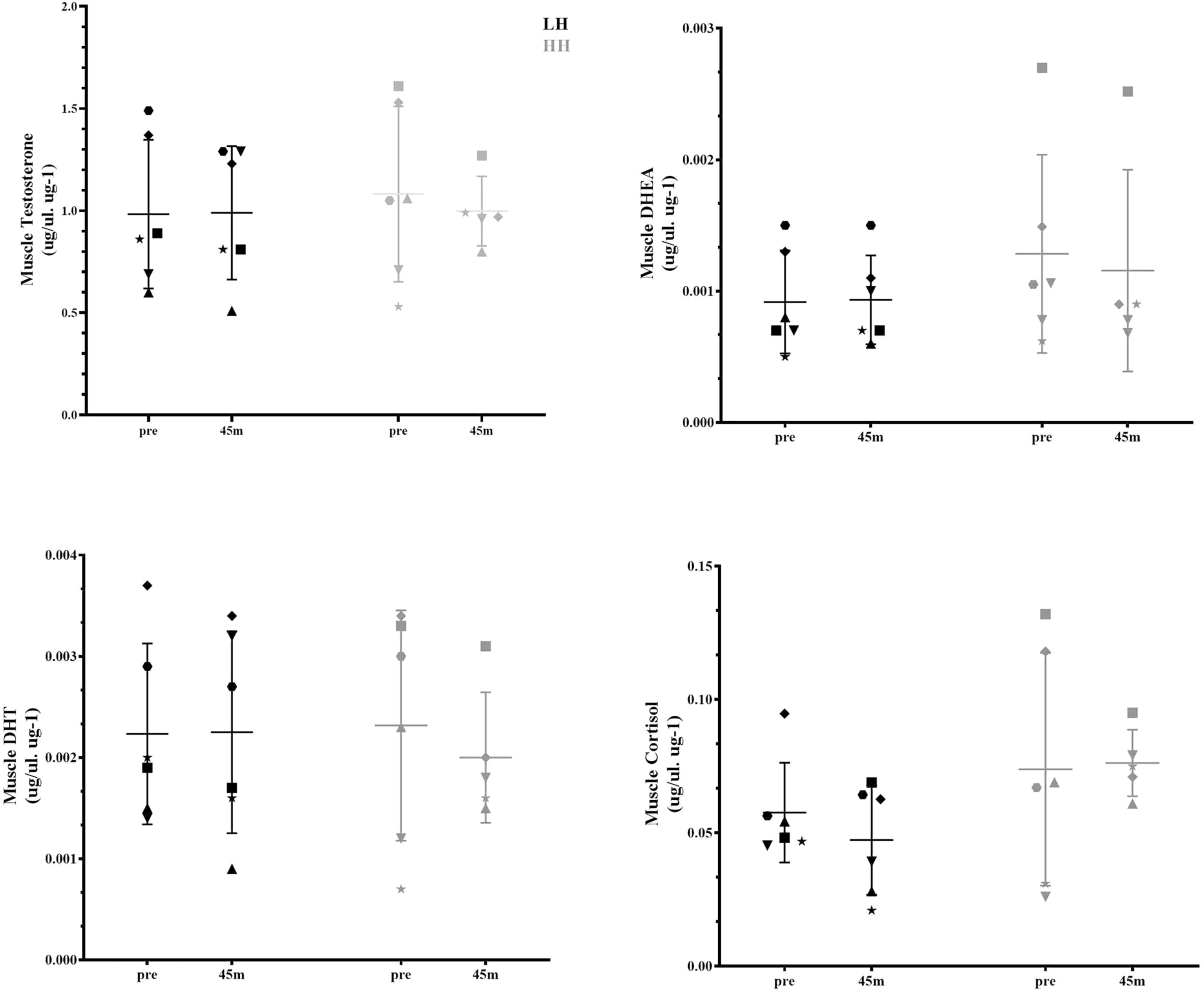
Muscle steroid hormone concentrations pre-LH and HH protocols and 45 min postexercise. Values are presented as the mean and SD. Each symbol represents one subject.

Serum cortisol significantly increased at 15 and 30 min after the HH protocol (*P* = 0.0006; *P* = 0.0020), with greater concentrations for the HH protocol than the LH protocol at the corresponding postexercise time points (*P* = 0.0217; *P* = 0.0335) (**Figure 1**). There were no significant changes in other serum steroid concentrations (**Figure 1**) or in muscle steroid concentrations after either protocol (**Figure 2**).

**Figure 3:**
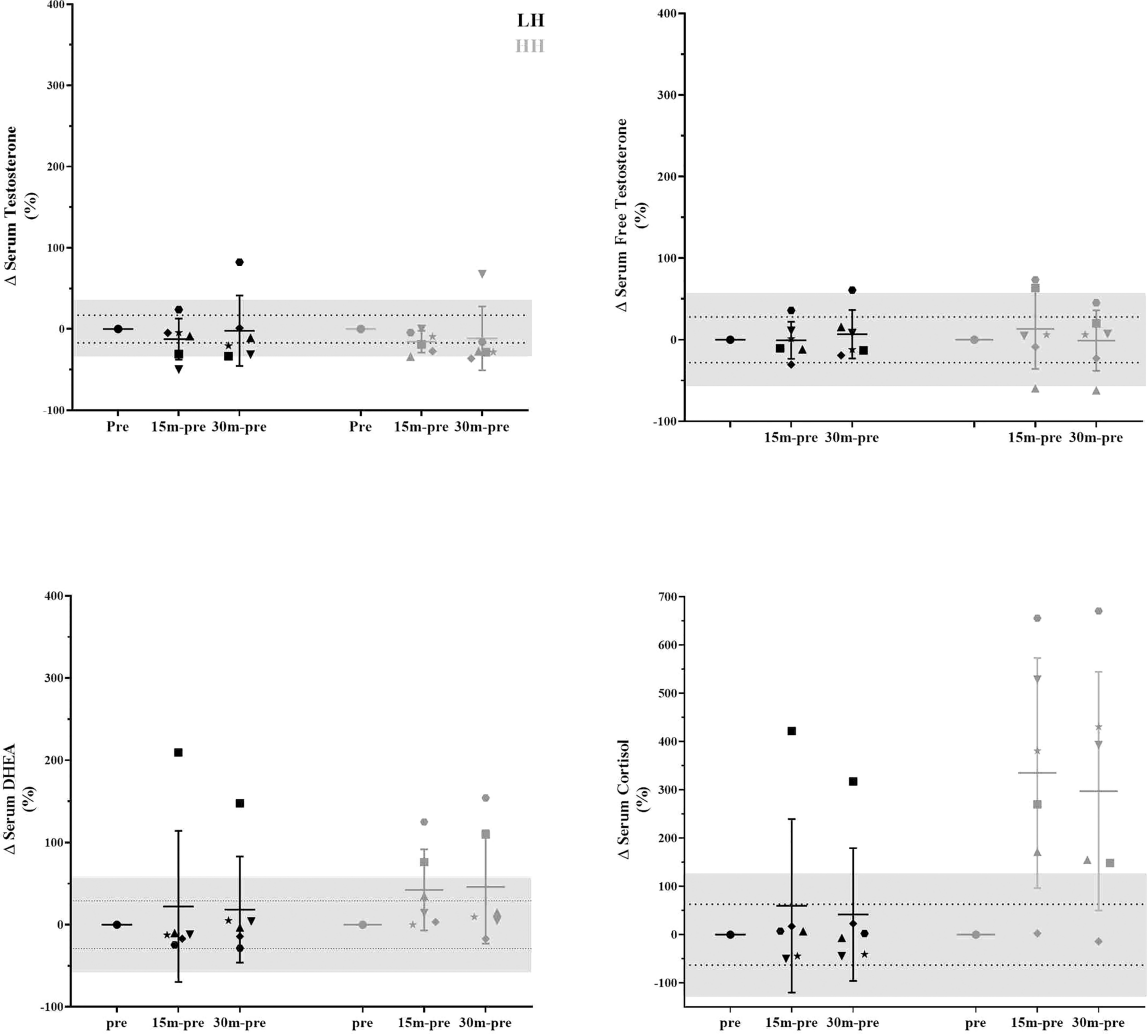
Delta changes in serum steroid concentrations pre-LH and HH protocols and at 15 min and 30 min postexercise. The gray area represents two times the typical error (positive and negative), and the dotted lines represent the typical error (positive and negative). Each symbol represents one subject.

**Figure 4:**
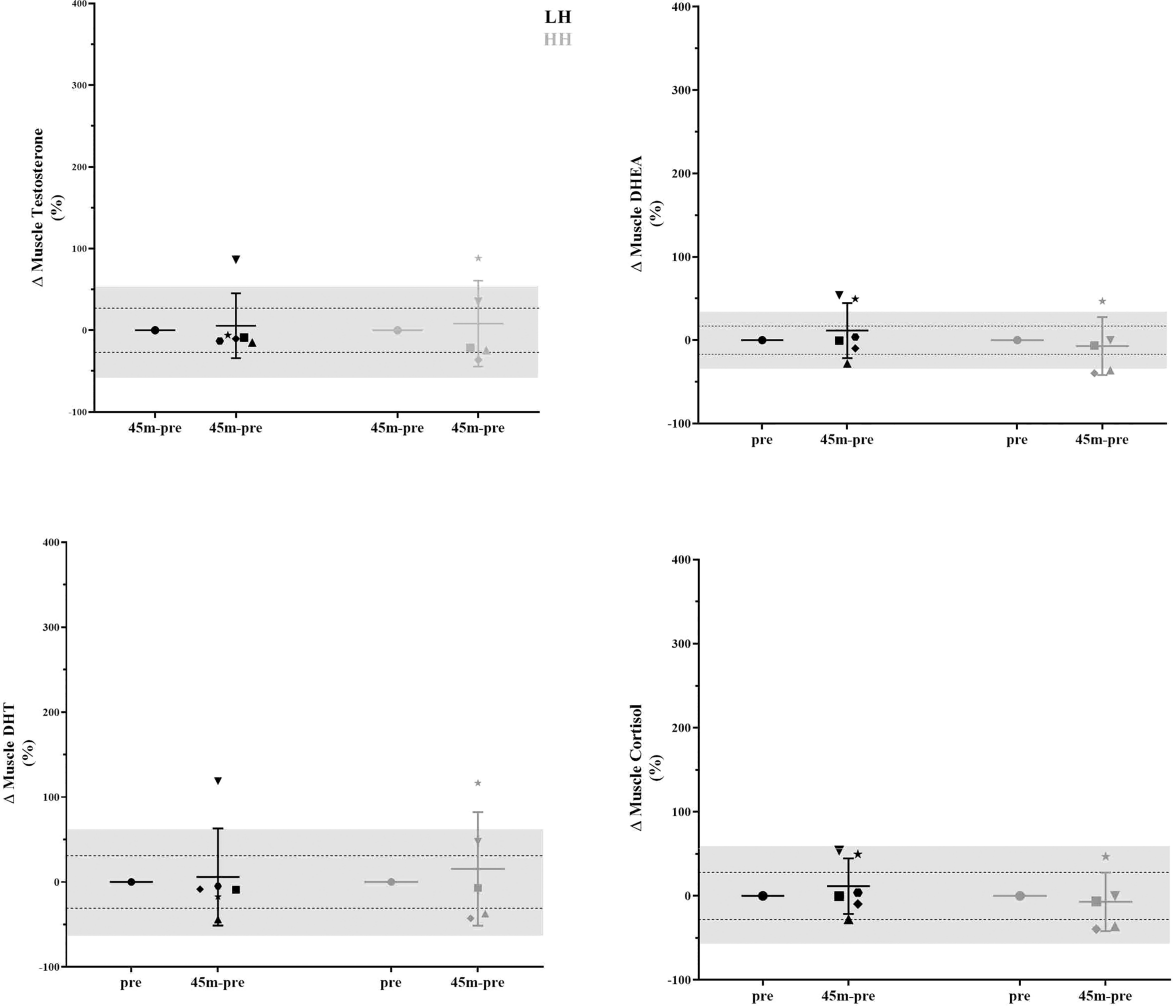
Delta changes in muscle steroid concentrations pre-LH and HH protocols and 45 min postexercise. The gray area represents two times the typical error (positive and negative), and the dotted lines represent the typical error (positive and negative). Each symbol represents one subject.

The intrasubject CVs (between baseline 1 and baseline 2) for serum concentrations of total and free testosterone, DHEA, and cortisol were 17% and 28%, 29%, and 63%, respectively. The muscle concentrations of testosterone, DHEA, DHT and cortisol intrasubject *CVs* (baseline 1 and baseline 2) were 27%, 17%, 31% and 28%, respectively. To verify whether the delta changes in serum and muscle steroid concentrations of each participant were higher than the measurement error (1x*CV* and 2x*CV*), we plotted the delta changes (**Figures 3**, **4**). For serum cortisol, 83% of subjects presented an increase two times higher than the *CV* at 15 and 30 min after the HH protocol. It is noteworthy that except for serum cortisol, all other changes in serum and muscular steroid hormone concentrations were within *2x CV*.

## Discussion

We aimed to determine whether the RE-induced increase in serum steroids was accompanied by elevated steroid concentrations in skeletal muscle tissue. Our main finding was that the increase in cortisol serum concentration did not align with the changes in the muscle steroid concentrations. In the present study, only serum DHEA and cortisol changed above 1x*CV* (29%) and 2x*CV* (126%) after the HH protocol, respectively, with no changes in any muscle steroid concentrations. Additionally, RE-related contractions of the deltoid muscle were not sufficient to change steroid concentrations in the muscle, although the volume and intensity of the LH protocol were high for the mentioned muscle (10 sets of 8 to 12 RM - 3 min rest interval between sets). The lack of change in muscle steroid after the LH and HH protocols suggests that individuals with previous experience in RE were desensitized to the exercise stimuli (serum and local) (11); it is also possible that the single postexercise timepoint investigated in this study (45 min after RE) to assess muscle steroid hormones might be too early or too late to observe any such changes.

Muscle steroidogenesis capacity in resistance-trained athletes has been investigated previously. Vingren, Kraemer (19) showed no changes in the concentration of testosterone in skeletal muscle at 10 and 70 min after RE despite a robust acute RE-induced increase in serum total and free testosterone concentrations (2). However, the large interval between these two time points and the lack of data for other steroids could explain their findings. Considering that in blood, RE-induced increases in steroid concentrations subside by 30 min after an exercise bout, hormones may have been absorbed by the target tissues or metabolized in the liver (22). As a result, the points (10 and 70 min after an RE bout) used by Vingren, Kraemer (19) may not best reflect changes in muscle steroid concentrations. We therefore assessed muscle steroid concentrations (i.e., testosterone, DHEA, DHT and cortisol) 45 min after the LH and HH protocols. Although we induced an increase in serum cortisol concentration, no changes were observed in muscle cortisol concentration or any other assessed steroid hormone. Additionally, our LH group allowed us to determine if muscle contractions *per se* (without increases in serum steroid concentrations) increase muscle steroid concentrations, as suggested by an *in vitro* experiment (23). Again, no changes were observed in any steroid concentration in skeletal muscle. Thus, additional timepoints should be examined to determine if RE can indeed change muscle steroid concentrations either by skeletal muscle uptake of these hormones or the intramuscular steroidogenesis process.

Although there was a lack of change to mean values (other than serum cortisol), we further scrutinized our data using individual values, which provided interesting observations. In **Figure 3**, in the LH protocol, the subject represented by the inverted triangle presents serum hormone concentrations always inside the measurement error. With close attention to the steroid concentration in skeletal muscle in **Figure 4**, the same subject is two times above the measurement error for testosterone, DHEA, and DHT and close to two times above the measurement error for cortisol. For this subject, since he did not present changes in serum hormone concentrations, it could suggest that a muscle contraction per se was able to modulate steroid hormone concentrations in skeletal muscle. In a similar direction considering the HH protocol, the subject represented by the star, also in **Figure 3**, presents serum hormone concentrations always inside the measurement error except for cortisol. For this subject, hormone concentrations in the skeletal muscle in **Figure 4** are two times above the measurement error for testosterone, DHEA, and DHT and close two times above the measurement error for cortisol. Remarkably, this subject did not present elevated steroid hormone concentrations in skeletal muscle (**Figure 4**) in the LH protocol (except for DHEA - > *2x CV*). Thus, the changes observed in testosterone, DHEA, DHT and cortisol with the HH protocol for this subject (represented by the star in **Figure 3**) could be due to altered serum cortisol concentrations (**Figure 3**) in addition to muscle contraction-induced DHEA concentration changes in the LH protocol (**Figure 4**).

Finally, the present findings should be interpreted taking into consideration the following factors: a) although the sample size is small in the present study (n=6), this is the first design presenting measurement errors (coefficient of variation and typical error) for serum and muscle steroid concentrations in humans; b) the methods to determine hormone concentrations should have very high sensitivity, as the muscle concentration of steroid hormones is extremely low. Thus, liquid chromatography/electrospray tandem mass spectrometry (LC–MS/MS) analysis has been suggested for these assessments (24). c) Possibly, we have lost the RE-induced increase in serum testosterone once 15 min after exercise, which can be too late to detect this increase (2). d) Considering that the RE protocols induced serum changes at least in cortisol, we cannot rule out the hypothesis that serum changes can affect muscle steroidogenesis, such as steroidogenic enzyme concentrations and activity. Thus, future works should investigate other muscle cell steroidogenesis-related alterations induced by cortisol. Additionally, serum changes in other steroids should be ensured to investigate steroid concentration and steroidogenesis in skeletal muscle. In conjunction, it seems necessary to conduct a time-course investigation of RE bout-induced changes in the concentration of muscle steroids and steroidogenesis. In conclusion, the serum steroid concentration increases (cortisol only) do not seem to be aligned with intramuscular steroid concentrations in previously resistance-trained men. Additionally, muscle contraction per se apparently cannot modulate steroid hormone concentrations in skeletal muscle.

## Data availability statement

The raw data supporting the conclusions of this article will be made available by the authors, without undue reservation.

## Ethics statement

The studies involving human participants were reviewed and approved by Ethics Committee of human research of the School of Physical Education and Sport (University of São Paulo, Brazil). The patients/participants provided their written informed consent to participate in this study.

## Author contributions

FCV, JV, GT, MC, CAL and CU contributed to conception and design of the study. FCV, GT, MC, ML, and FD collected the data. LR proceeded the muscle biopsies. FCV, GT, TC, and PB proceeded the biochemical analises. FCV organized the database. FCV and CU performed the statistical analysis. FCV wrote the first draft of the manuscript. All authors contributed to manuscript revision, read, and approved the submitted version.
